# Lymphocytes in the neighborhood: good or bad for the kidney?

**DOI:** 10.1172/JCI160657

**Published:** 2022-07-01

**Authors:** Hao Li, Maria G. Tsokos, George C. Tsokos

**Affiliations:** Department of Medicine, Beth Israel Deaconess Medical Center, Harvard Medical School, Boston, Massachusetts, USA.

## Abstract

Lupus nephritis (LN) is common in people with systemic lupus erythematosus (SLE) and advances, almost invariably, to end-stage renal disease (ESRD). In this issue of the *JCI*, Abraham, Durkee, et al. presented a large-scale immune cell landscape of kidney biopsies from patients with LN by combining multiplexed confocal microscopy imaging with customized computer vision and quantification. The presence of diverse CD4^–^ T cells in small neighborhoods, but not of B cells or CD4^+^ T cells in large neighborhoods, is linked to the development of ESRD. Unexpectedly, B cells in the kidney heralded a good prognosis. The precise location of different types of immune cells allows inference on possible interactions between different immune cells and also between immune and kidney-resident cells. The data have important implications on the development of prognostic tools and effective targeted therapies in patients with LN.

## Lupus nephritis and computational pathology

Lupus nephritis (LN) is characterized by immune complex deposition and the presence of immune cell aggregates in kidney tissue, which result in inflammation, injury, and irreversible damage (1, 2). Kidney biopsy represents the definitive tool to diagnose and evaluate histologic and immunologic features of LN (3). Although the management of patients with LN has improved during the last several decades, the risk to develop end-stage renal disease (ESRD) remains largely unchanged. Clearly, early prediction of ESRD and prompt initiation of specific treatment are of vital importance to improve outcomes for patients with LN.

Although patients with LN frequently present with circulating dsDNA antibodies and low complement levels (4) and have distinct urine findings — including protein and cellular casts — it is the inflammatory response within the kidney parenchyma that is closely associated with the development of ESRD (5, 6). The dysregulated immune response in patients with LN — determined by examination of immune cells in the peripheral blood — may have little, if any, relevance to the events in the inflamed kidney (1, 7). It appears that the cellular organization of the inflammatory response, as studied in this issue of the *JCI* by Abraham, Durkee, et al. (8), may better predict progression of renal disease and clinical outcome in patients with LN. The authors provided a cutting-edge approach to mine clinically useful information by immunomapping the kidney. Recent single-cell transcriptome analyses of immune cells isolated from LN-kidney biopsies have provided ample information on the nature and abundance of infiltrating cells (9, 10). Yet, these data are inherently unable to provide information regarding the physical relationship between (a) the examined immune cells and (b) the immune and kidney-resident cells. More importantly, the behavior of the immune cells may be dictated by metabolites or chemokines produced by kidney-resident cells. For example, tubular epithelial cells exposed to IL-23 do not metabolize arginine, which is released in the interstitium, leading to lymphocyte proliferation (11). Similarly, local hypoxia enables infiltrating T cells to resist death (12).

Artificial intelligence-based (AI-based) computational pathology is an emerging discipline that has recently shown great promise to improve diagnostic efficiency and accuracy in oncology (13). Abraham, Durkee, et al. (8) applied computational approaches on biopsies of chronically inflamed kidneys from patients with LN and demonstrated that there are distinct in situ inflammatory neighborhoods that are characterized by variable cellular densities of immune infiltrates. Furthermore, the authors have provided a detailed association between in situ inflammatory areas and disease outcome, while highlighting the clinical value of computational pathology, which offers a different strategy to predict ESRD in lupus nephritis.

## In situ inflammatory neighborhoods in LN

Abraham, Durkee, et al. (8) studied patients who had 2-year minimum follow ups. Biopsy samples were spatially assessed to establish the immune cell landscape and analyzed for their ability to predict the development of ESRD. Because cell-cell contact is key to immune cell communication, the authors defined regions of interest (ROIs) by the presence of T cells and quantified 5 types of immune cells: B cells, CD4^+^ T cells, CD4^–^ T cells, plasmacytoid dendritic cells (pDCs), and myeloid dendritic cells (mDCs). Although the total cell count was higher in the ESRD^+^ cohort, the overall cell densities in ROIs were similar. Notably, though, the differences between B cell and CD4^–^ T cell densities in patients with and without ESRD were robust. Higher densities of B cells were associated with lower tubulointerstitial inflammation chronicity scores and better prognosis, while an opposite trend was observed for CD4^–^ T cells. Neither the densities of CD4^+^ T cells nor those of pDCs were different between the 2 groups of patients. Among the patients who developed ESRD, there was a small subset who were already in renal failure at the time of biopsy, and those patients had fewer B cells and a more apparent increase of CD4^–^ T cells compared with patients who were not in renal failure. Additionally, a profound depletion of mDCs was observed exclusively in this small cohort of patients in renal failure. Well-circumscribed immune cell aggregates are frequently observed in kidney biopsies of patients with LN, and their organization has been studied (5, 6, 14). Abraham, Durkee, et al. (8) showed that both B cells and CD4^–^ T cells displayed a strong tendency to interact and congregate with like cells, explaining the presence of distinct cell neighborhoods dominated by either B or CD4^–^ T cells.

## Immunopathogenesis of LN

**Figure 1:**
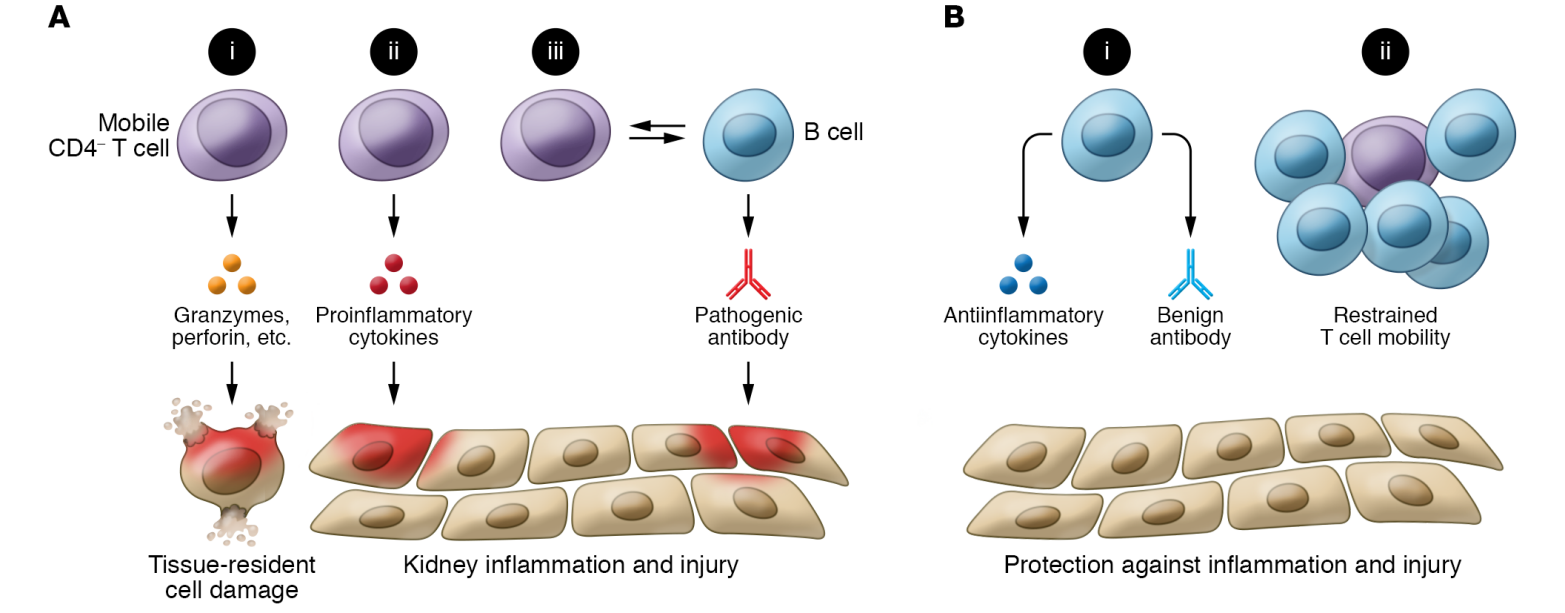
Kidney-infiltrating lymphocytes predict injury or protection. (**A**) Renal CD4^–^ T cells may induce kidney damage via 3 possible pathogenic pathways: (i) cytolytic killing, (ii) production of proinflammatory cytokines, and (iii) interaction with B cells to produce pathogenic autoantibodies. (**B**) Renal B cells may protect against renal inflammation and injury via 2 potential mechanisms: (i) release of immune-regulatory factors and (ii) establishment of a microenvironment to restrain immune cell mobility. Neighborhoods without protective B cells that are enriched with CD4^–^ T cells retain a mobility-promoting microenvironment.

Systemic lupus erythematosus (SLE) is an autoimmune disease characterized by (a) the production of autoantibodies targeting cellular and nuclear components and (b) immune complex deposition in various organs, which mediates tissue inflammation, injury, and damage (15). The contribution of B cells to the pathogenesis of SLE has long been considered a central mechanism, and researchers have entertained B cell–targeting approaches to treat patients with this disease (16). It is surprising and also interesting that the presence of large cell neighborhoods enriched with B cells predict the preservation in kidney function. There are a number of potential explanations for the protective role of B cells in the kidney: (a) B cells infiltrating the kidney in patients with LN may have a regulatory function; (b) B cells may have been called there to contain inflammation either through direct cell contact or through the production of regulatory cytokines, such as IL-10 (17); (c) they may adsorb locally produced autoantigens without presenting them to T cells; or (d) they may establish a distinct microenvironment in which they sequester pathogenic T cells to block their action (Figure 1). These possibilities warrant future investigation; specifically, spatial transcriptomics and metabolic studies would shed light on the protective role of B cells in preserving kidney function and could explain the universal lack of clinical effect of B cell depletion in patients with LN.

A strong association between the presence of CD4^–^ T cell populations and the high risk of progression to renal failure was identified. In contrast to B cells, these CD4^–^ T cells preferentially formed small cellular neighborhoods of less than 20 cells. The authors pointed out that the CD4^–^ population was heterogeneous and the presence of these cells heralded substantial renal injury (8). In agreement with previous studies — with the exception of γδ and CD8^+^ αβ — a substantial number of CD4^–^CD8^–^ αβ (double negative [DN]) T cells were observed, and these cells are known to arise from CD8^+^ self-reactive T cells (18, 19). The capacity to produce various inflammatory cytokines and the cytolytic potential of these cells make them putative key contributors to the renal injury (Figure 1) (20). Although the interaction between B and CD4**^+^** T cells is crucial for optimal antibody responses, the aberrant help provided to B cells from DN T cells for autoantibody production in SLE cannot be ignored — especially when noncharacterized T cells, but not T follicular helper cells, were found near B cells (Figure 1) (21). It is still unclear whether CD4**^+^** cells enter the kidney after they have been stimulated in the periphery or whether they enter as naive cells responding to an inviting hypoxic, nutrient-rich local milieu (11, 12). The demonstration that T cells predict poor renal function outcome (8) suggests a pathogenic role executed through their ability to produce IL-17 (18) or direct cytotoxic activity, and argues against the claim that the infiltrating cells lose their pathogenic potential (22). Parallel peripheral blood and infiltrating T cell receptor repertoire analysis should provide insight into this question. In addition, concerns about lymphocyte access to kidney tissue should be addressed. An intact glomerular basement prevents cytotoxic cells from entering the glomerular tuft and destroying podocytes (23). Consequently, events that enable the entrance of immune cells into the kidney parenchyma become especially important.

A key issue in addressing the pathogenesis of LN is the interaction between immune and resident cells. Abraham, Durkee, et al. (8) report that CD4^–^ T cell neighborhoods reside in closer proximity to tubules than B cell neighborhoods, suggesting that cellular neighborhoods are spatially organized. Although we could infer that cross-talk occurs between immune and resident cells at this point, in situ transcriptomic and metabolomic analysis and advancing technologies can now inform us about how immune cells impact the function of resident cells and vice versa. Injured podocytes express the costimulatory molecules CD80 and CD86 and major histocompatibility complex molecules (24), which implies that T cells crossing the basement membrane (23) may be stimulated locally. There is early evidence that kidney-resident cells may avert immune-mediated pathology. Lupus-prone mice that lack the calcium/calmodulin serine/threonine kinase 4 in podocytes (25) or in tubular epithelial cells (11) do not develop glomerulonephritis. It is possible that the observed effects of T cells and B cells — damaging and protective, respectively — may reflect the effect of other factors, including genetics or input from tubular epithelial cells and/or podocytes.

## Conclusions

The heterogeneity of the immune landscape of LN means that singular treatments are unlikely to benefit all patients and confirms the need for personalized approaches. Kidney biopsy plays a crucial role in the diagnosis on specific forms of LN, but the prognostic value of qualitatively evaluating an in situ inflammatory response remains under appreciated. Abraham, Durkee, et al. (8) provide evidence that the nature of the immune cells that infiltrate the kidney in patients with LN is important in predicting ESRD. Advanced AI technologies should make it easier for such information to enter clinical practice. Some patients may benefit from preventing T cell entry in to the kidney and some from enabling the entry of B cells. We eagerly await studies that confirm the data in larger cohorts.

## Version 1 07/01/2022

Electronic publication
